# New Iodine Food Composition Database and Updated Calculations of Iodine Intake among Norwegians

**DOI:** 10.3390/nu10070930

**Published:** 2018-07-20

**Authors:** Monica H. Carlsen, Lene F. Andersen, Lisbeth Dahl, Nina Norberg, Anette Hjartåker

**Affiliations:** 1 Department of Nutrition, Institute of Basic Medical Sciences, University of Oslo, 0372 Oslo, Norway; l.f.andersen@medisin.uio.no (L.F.A.); nina.norberg@medisin.uio.no (N.N.); anette.hjartaker@medisin.uio.no (A.H.); 2 Institute of Marine Research, P.O. Box 1870 Nordnes, 5817 Bergen, Norway; lisbeth.dahl@hi.no

**Keywords:** iodine, dietary intakes, food composition

## Abstract

Iodine food composition data of Norwegian foods have been sparse and knowledge about different dietary iodine sources limited. We compiled a comprehensive iodine food composition database and estimated dietary iodine intake among adults in the latest Norwegian national dietary survey (Norkost 3). The iodine content of food and beverages were compiled using international guidelines and standards. Iodine content of 3259 food items were compiled, including analytical values, values from other food composition databases, estimated values, and values that were based on recipes. Estimated iodine intake in the Norkost 3 population ranged from 15 to 1462 µg/day. Men had significantly higher intake of iodine than women (*p* < 0.001). The proportion of men and women with estimated iodine intake below average requirement was 19% and 33%, respectively. In young women, 46% had estimated iodine intakes below average requirement and a high probability of inadequate iodine intake. Several dietary sources contributed to iodine intake and differences in the consumption pattern may put subgroups at risk of insufficient iodine intake. In the coming years, the determination of iodine in foods and national dietary surveys should be regularly performed to monitor the iodine intake in the Norwegian population.

## 1. Introduction

Iodine is an essential nutrient for the synthesis of thyroid hormones, which are involved in regulating the body’s metabolism and they are required for normal growth and the development of the brain and central nervous system [1,2,3]. Iodine deficiency in humans results in a variety of disorders depending on the severity of the deficiency and at what age the deficiency occurs [1,3]. Sufficient iodine intakes are of pivotal importance during pregnancy and early childhood. Later in life, inadequate levels of iodine may result in goiter and other adverse health outcomes [1,3]. On the other hand, excessive iodine intake has been associated with increased rates of thyroid dysfunction [1,4]. Since both iodine deficiency and excess iodine exposure are associated with adverse outcomes, assessing population iodine status is an imperative public health initiative. To assess iodine status in a population, measurement of urinary iodine concentrations (UIC) is recommended, while estimations of intake may be done using dietary assessment methods and food composition data [5,6].

Historically, studies conducted in Norway between 1914 and 1938 showed high prevalence of goiter, especially among people living in inland areas where intakes of fish were low. In some inland areas, almost 80% of schoolchildren had goiter [7,8]. Preventive measures were implemented using voluntary salt iodization (5 µg/g). Further, iodine enriched cattle fodder was introduced, which resulted in dairy products with elevated iodine concentrations. Follow-up studies performed in the 1970s and 1980s showed a marked reduction in goiter [7,8]. Up until now, no studies estimating iodine intake in both genders have been conducted in Norway. Recent studies in pregnant women suggest that the iodine intake in the general population has changed and may now be too low [7,8,9,10,11,12,13]. The worldwide recommended strategy to secure sufficient iodine intake in a population is implementation of Universal Salt Iodization Programs [2,13]. In Norway, neither industrial nor household salt have mandatory iodization.

People obtain iodine from the diet, their drinking water, and dietary supplements [14], therefore, all dietary iodine sources are of importance when assessing total intake. Iodine is among the micronutrients with the largest variation in concentrations in foods; between different food groups, as well as between otherwise similar food items; i.e. between and within different species of seawater fish [14,15,16]. This variation is due to several factors; as most of the earth’s iodine is in the seas, fish and seafood generally have high content of iodine and so have plant foods that are grown near the coast, as compared to plant foods grown in inland areas [14]. In addition, changes in the environment in which fish live and in the processing of fish, changes in soil, use of fertilizers, changes in the composition and fortification of cattle and chicken fodder, and use of iodized salt in food processing, all influence the total iodine content in our diets [14,15,16]. Furthermore, changes in food habits may influence the dietary intake of iodine and individuals whose diets exclude or restrict iodine-rich food sources, for health, religious, or other reasons are at a special risk of deficiency [17]. 

Both the international research community [15,17,18] and the Nordic Nutrition Recommendations of 2012 [19] underline the need for better surveillance and more data on the level of iodine in foods. Studies in nutrition epidemiology are dependent on high quality food composition databases to make good estimations of intake. The reliability of estimates of iodine intake relies on the quality of the iodine food composition data [20] and reliable and representative iodine food composition data is therefore an important prerequisite [15,21]. Comprehensive and high quality data on food iodine content have been sparse; values have been lacking or have been obsolete [3]. However, initiatives have recently been taken to fill the missing data gap and analytical food iodine content data are now emerging [17,18]. Analysis of iodine in food and beverages has historically not been included in the Norwegian analytical food composition projects and has until recently only been done in a limited number of foods. Thus, there has been limited iodine food composition data available for Norwegian foods. This is, however, changing and recent analytical food composition projects now include determination of iodine. 

In the present project, our first aim was to compile and update the iodine food composition databases at the University of Oslo and the Norwegian food composition table (matvaretabellen.no). The second aim was to use these data together with the latest national dietary survey among adults, in order to estimate the populations’ dietary iodine intake in both men and women and in different age groups.

## 2. Materials and Methods 

### 2.1. Compiling of Iodine Food Composition Data

The compiling of iodine values for the food composition database and nutrient and food calculation system “Kostberegningssystem” (KBS) at the University of Oslo, was conducted following the guidelines for food composition compiling, as described in “Food composition data, production, management and use”, by Greenfield and Southgate [20]. In addition, the food composition compiling and management tool from FAO/Infoods, “Compilation tool version 1.2.1” (FAO/INFOODS) and its user guidelines were used [22]. Data compilation was performed in the period April 2017 to March 2018. Analytical values from Norwegian analytical projects, seven projects conducted in the period 2010 to 2017, three projects conducted in 1996, 2000, and 2002, respectively, and food composition data from international scientific food research articles, were the main sources of iodine food values. When this was not available, iodine values from other food composition databases were used, according to the standard compilation procedures [23,24]. When compiling from scientific literature and food composition databases the most important factors considered were the matching of the food item, if the food item had been fortified with iodine or not, and the time since the analysis was done; using the most recent data available.

### 2.2. Data Sources

Data was compiled from analytical projects planned and conducted by the Norwegian Food Safety Authority (NFSA) for the purpose of generating representative and objective food composition data for the Norwegian Food Composition Database and by the Institute of Marine Research (IMR) in Norway. In addition, iodine values from the food composition databases of the Swedish National Food Agency (www7.slv.se) [22]; the Danish DTU Food, National Food Institute (Fooddata rel. 2 and 3) [25]; the Finnish National Institute for health and welfare (Fineli database) [26]; the Dutch National Institute of Public Health and Environment (NEVO online version 2016/5.0) [27]; the English McCance and Widdowson’s composition of foods integrated dataset [28]; and, the Australian AUSNUT 2011–2013 AHS Food Nutrient Database [29], were compiled. Furthermore, iodine values were compiled through literature searches on PubMed, Medline, Web of Science and Google Scholar, using search terms “iodine”, “iodide”, “food”, and/or “composition”, identifying scientific articles that presented analytical iodine food composition data. For dairy products, analytical values were also compiled from TINE SA (Oslo, Norway), the major Norwegian dairy company. 

The iodine content of composite dishes was calculated based on recipes in the KBS database, version AE14. 

All of the original iodine values were saved unaltered in an archival database, and subsequently matched to the food items of the AE14 food composition database in KBS, according to the guidelines and standards of FAO/Infoods food composition compiling procedures [23,24,30]. 

### 2.3. Study Population Norkost 3

The latest national dietary survey, Norkost 3, was conducted in 2010–2011 using two 24 h dietary recalls [31]. A total of 4847 people, aged 18 to 70 years, were randomly drawn from the National Registry at the Norwegian Tax Administration and were invited to the study by a postal invitation letter together with an information pamphlet. Of those invited, 2148 declined the invitation, 147 later withdrew their consent, 235 did not complete the study, and 530 did not answer the invitation. Thus, 1787 men and women completed the dietary survey. After the participants gave their consent and were included in the study, 24 h recall interviews were conducted over the phone twice, with an interval of approximately four weeks between the first and second interview. The interviews were conducted by trained interviewers at the Department of Nutrition, University of Oslo, in the period of January 2010 to August 2011 [31]. All of the nutrient intakes were calculated in KBS, at the University of Oslo (Oslo, Norway). All of the subjects gave their informed consent for inclusion before they participated in the study. The present study was conducted in accordance with the Declaration of Helsinki, and the protocol was approved by the Regional Committees for Medical and Health Research Ethics (REC South East, project code 2009/1318b). Further details on the Norkost 3 survey are given in Totland et al. [31] and Myhre et al. [32].

### 2.4. Statistical Analyses

The distribution of the data was evaluated using histograms, normal Q-Q (quantile-quantile)-plots, and the Shapiro-Wilk test. Normally distributed data are presented as mean and standard deviation (SD), non-normally data are presented as medians and 25th and 75th percentiles, if not otherwise stated. To test for differences in the intakes between men and women, the non-parametric independent samples Mann-Whitney U test was used. To test for differences between age groups within a gender the non-parametric Kruskal-Wallis test was used. Significant differences were set to *p* < 0.01. 

## 3. Results

### 3.1. Iodine Food Composition Data

In this project, we compiled iodine values for 3259 food items, beverages, and supplements.

#### 3.1.1. Milk and Dairy Products

A total of 287 dairy products were assigned iodine values. Due to recent changes in iodine fortification of cow fodder in Norway (personal communication Leidulf Nordang, Felleskjøpet, and Trøan et al. 2018 [33]) only analytical data from 2016 and 2017 were used when compiling values for the most commonly consumed milk types (whole milk, semi-, and skimmed milk). For other dairy products, the most recent analytical values available were used, although, some of these originated from before 2015. In the food categories milk, cream, sour cream, and milk-based ice cream, 42 items were assigned analytical values, 32 were estimated based on similar food items, and 112 items were assigned values from recipes. The mean iodine content in milk and cheese are presented in Table 1. The mean value of 16 µg/100 g for milk includes values for both conventional and organic milk and all the categories of milk fat content. With regard to cheese, of a total of 101 food items, 28 were assigned values from Norwegian analytical projects, 29 were estimated based on similar products, 27 were assigned values borrowed from other food composition databases, and 17 were assigned values based on recipes. The iodine values for Norwegian white and whey cheese were compiled from analytical projects. Whey based cheese had a 7.5-fold higher concentration of iodine than white cheese that is based on cow milk. Cheese made from goat milk had on average higher iodine content than cheese based on cow milk.

#### 3.1.2. Fish and Seafood 

A total of 255 fish products, including raw fish, cooked fish, and raw and heat treated seafood and other fish products were assigned iodine values. Of these, 44 items were assigned analytical values (data from IMR), 27 were assigned values from other food composition databases, and 184 items were assigned values either estimated from similar food items or calculated using recipes. Mean and range of iodine content of some important fish for human consumption are presented in Table 1, showing a large variation in iodine content in fish, between and within fish species. 

#### 3.1.3. Egg

Mean iodine content in egg, including raw, cooked, and fried eggs from both conventional and organic hen eggs are presented in Table 1. All of the raw egg items were assigned analytical values, whereas iodine content in egg dishes was calculated using recipes. 

#### 3.1.4. Bread

Iodine content in bread is presented in Table 1. Data on iodine in bread were compiled from Norwegian analytical projects (24 items), other food composition databases (2 items), and estimated from similar foods or calculated from recipes (136 items). 

#### 3.1.5. Meat

In total, 386 food items were assigned iodine values, of these 11 were analytical values, 62 were compiled from other food composition databases, and the remaining 313 food items were assigned iodine values from similar food items or recipes. 

#### 3.1.6. Fruit and vegetables

Iodine content in fruit and vegetables were compiled from analytical projects (two items), other food composition databases (119 items), or estimated from similar food items or from recipes (439 items). 

#### 3.1.7. Coffee

The food group “Coffee” in KBS includes different types of processed and ready-to-drink coffee drinks, including milk-based recipes. Of the 23 items in this group, six items were assigned iodine values from the Swedish Food Composition Database and iodine values for the remaining 17 items were estimated from similar products or calculated using recipes. 

#### 3.1.8. The Norwegian Food Composition Database

Iodine values compiled in the present study are presented in the Norwegian Food Composition Database, version 2018, published by NFSA (matvaretabellen.no).

### 3.2. Iodine Intakes in the Norkost 3 Population

Detailed characteristics of the study participants have been published earlier [31]. Summarized, the population consisted of 862 men and 925 women, aged 18 to 70 years. In men, mean age was 47 (SD 14.4) years and mean body mass index (BMI) 26.3 (SD 3.5) kg/m^2^. In women, mean age was 45 (SD 13.3) years and mean BMI was 24.6 (SD 4.2) kg/m^2^. Non-smokers amounted 79% of the population.

Intake of iodine in the Norkost 3 population showed a skewed distribution and ranged from 15 to 1462 µg/day, with a median of 151 µg/day, and a mean of 202 µg/day. Intakes of iodine in men and women, in different age groups, are presented in Table 2. 

Men had a significant higher intake of iodine than women (*p* < 0.001). In men, the median intakes across age groups, from young to old, ranged from 153 µg/day to 197 µg/day (*p* = 0.002). In women, the median intakes across age groups ranged from 110 µg/day to 159 µg/day (*p* < 0.001) (Table 2). Of the male participants, 18% had high probability of inadequate iodine intake (estimated intakes below average recommendation (AR), defined as 100 µg/day). For females, this percentage was 33% for all women and 46% for women age 18 to 29 years (Table 2). In men, 7% had very high probability of inadequate intake (estimated intakes below lower intake lever (LI), defined as 70 µg/day). In women, 13% had very high probability of inadequate intake, and for women aged 18 to 29 years, this proportion increased to 24%. Approximately 7% of the men age 50 to 70 years had an intake above Upper Intake Level (UL, 600 µg/day). Very few women had an intake of iodine above UL (Table 2). 

### 3.3. Sources of Iodine in the Norwegian Diet

Table 3 and Table 4 present the sources of iodine in the total diet of men and women, respectively. 

In men, milk, cheese, and other dairy products and fish and seafood contributed 37% and 22% of total iodine intake, respectively (Table 3). Coffee contributed 11%, and bread, cereals, cakes, fruit, berries, vegetables, potatoes, egg, and meat together contributed approximately 19% of the total iodine intake. The young men, age group 18 to 29 years, had a higher contribution of iodine from bread, cereals and cake, meat, dairy products, milk, white cheese, and mineral and drinking water as compared to the older men, and a lower contribution from total fish and seafood, lean fish, fatty fish, as well as from whey cheese, total intake of beverages, and coffee (Table 3). The iodine intake presented in Table 3 accounts for 95% of the total iodine intake in men in Norkost 3. The remaining 5% came from sauces, condiments, snacks, chocolate, and butter. Among men, 5% had iodine intakes above UL, and in this group, on average, 76% of the iodine intake came from fish.

When including consumers only, men who consumed lean fish (*n* = 210) got on average 58% of their dietary iodine from lean fish and those who consumed whey cheese (*n* = 275) got on average 17% of their iodine intake from whey cheese (Table 3). Supplements contributed to on average 4% of total iodine intake among all men and 34% of total iodine intake in male supplements users. 

In women, milk, cheese, and dairy products and fish and seafood intake contributed 34% and 20% of total iodine intake, respectively (Table 4). Coffee contributed 12%, while the sum of bread, cereals, cakes, fruit, berries, vegetables, potatoes, meat, and egg together contributed 20% of total iodine intake. Women, aged 18–29 years, had a higher average contribution of iodine from bread, cereals and cakes, meat, dairy products, milk, white cheese, and mineral and drinking water as compared to the older age groups. However, they had a lower contribution of iodine from total fish and seafood, lean fish, egg, total beverages, and coffee as compared to the older women (Table 4). The iodine intakes that are presented in Table 4 accounts for 95% of the total iodine intake in women in Norkost 3. The remaining 5% came from sauces, condiments, snacks, chocolate, and butter. 

When including consumers only, women who ate fish (*n* = 581) got on average 32% of their iodine intake from fish, women who consumed lean fish (*n* = 220) got on average 58% of their dietary iodine from lean fish and those who consumed whey cheese (*n* = 317) got on average 18% of their iodine intake from whey cheese. In addition, coffee-drinkers got 15% of their iodine from coffee (Table 3). Supplements contributed to, on average, 6% of total iodine intake among all women and 39% of total iodine intake in female supplements users. 

Men had a higher contribution of iodine from meat, total intake of dairy products, and milk than women (all *p* < 0.01), while women had a higher contribution of iodine from fruit, berries, nuts and seeds, yoghurt, mineral and drinking water, and supplements, than men (all *p* < 0.01)

## 4. Discussions

We have compiled a comprehensive database on the iodine content of Norwegian foods, from analytical projects, other food composition databases and scientific food composition literature. Men were in better compliance with the iodine recommendations than women, yet nearly one in five males had high probability of inadequate iodine intake. In women, almost a third had high probability of inadequate iodine intake, and especially among the younger women the iodine intake was low. The present study shows that other food groups in addition to lean fish, milk, and dairy products contribute considerably to the total iodine intake. The present study adds important new knowledge to an area with sparse data, regarding the total iodine intake and the contributions of the different dietary iodine sources of Norwegians.

The low intake of iodine in women in the present study is in agreement with earlier studies showing a low iodine intake among women in Norway [7,8,9,10,11,12,13]. The young women are at particular risk of inadequate iodine intake; only 41% had an iodine intake above recommended intake and among women age 18 to 29 years, 46% and 24% had high and very high probability of inadequate intake, respectively. Low iodine intakes in women in childbearing age have also been reported from other Nordic and European countries [8,33,34]. 

The iodine intake in both men and women showed great inter-individual variation, different iodine food sources, and varying intakes across age groups, which indicate that the Norwegian population depends on a variety of dietary iodine sources to fulfill their iodine needs. Young men and women in the present study had not only lower intakes of iodine than older participants did, but also other food sources. In young adults, the most important iodine sources were milk, meat, and white cheese, while in the older age groups, the foods contributing mostly to iodine intakes were fish and especially lean fish and fish products, in addition to whey cheese, milk, and coffee. This difference in iodine sources is partly explained by the differences in intake of fish and milk products seen between young and older age groups in the Norkost 3 survey [31] and by changes in the diet of the Norwegian population; while the intake of white cheese has increased, there has been a decrease in intakes of other milk products and fish [35]. Dietary changes will influence the iodine intake of the population and the present findings are in agreement with the study of Katagiri et al. [36], showing changing dietary patterns among younger men and women, resulting in lower intake of iodine as compared to the traditional Japanese diet. 

Coffee is, in the present study, identified as a source of iodine in the Norwegian population. This is a consequence of the traditionally high consumption of coffee in Norway [31]. Again, it is interesting to see a difference across age groups; the contribution of iodine from coffee is higher among the older participants of the population than among the young. 

The iodine intake increased with age for both men and women, but still 12% and 27% of men and women, respectively, in the age group 60 to 70 years, had a relatively high probability of inadequate intake. In the other end of the intake distribution, 5% of the men in this study had iodine intake above UL 600 μg/d [19], and this high intake was mostly due to iodine from fish. This upper intake level is however low when compared to the upper intake level defined for Americans at 1100 μg/day [37] and 3000 μg/day set by Japanese dietary guidelines [21]. 

There are several limitations with the dietary data used in the present study. The iodine recommendation refers to the average intake over a longer period of time [19]. However, the present iodine intake data was based on two 24 h recalls, which provides dietary data on group level, but it is somewhat inadequate for estimations of individual habitual diet [38]. Moreover, with two recalls, the intake of food items eaten infrequent or seldom most probably will be underreported. This may have implications for the estimations of iodine intake from fish in the present study, which may be underestimated.

To assess iodine status in a population, the WHO/UNICEF/ICCIDD recommend measuring UIC [2]. The present study did however not collect urinary samples and may therefore only estimate iodine intakes without the evaluation of UIC [5].

In Norway, table salt and salt used in food manufacturing are not fortified with iodine, and only a few commercially available table salts have iodine content (5 μg/g). The contribution of iodine from iodized salt is therefore very uncertain, but may contribute to the overall iodine intake in the population. 

In the present compilation project the iodine value in milk was based on a large number of individual samples that were analyzed by the TINE SA dairy company and the IMR. Based on scientific literature on the production of milk and its content of iodine, which showed no significant differences in iodine content based on milk fat content, we used the average value of iodine in all milk types, irrespective of fat content [39,40,41]. Furthermore, the development of new cattle fodder including soy-based ingredients has influenced the content of iodine in Norwegian milk recent years [42,43], and thus only analytical values for the two most recent years were included in our iodine food composition database. Not surprisingly, whey cheese had a much higher content of iodine than white cheese. Iodine is mostly found in the water fraction of milk (the whey), and thus it is concentrated in whey cheese. 

The iodine content of industrially produced bakery products may be higher than the values that we have compiled in this project. The reason is that between 2004 and 2016 at least 168 bread and other bakery products on the Norwegian food market, mostly imported products, were produced while using iodized salt in different concentrations, according to the NFSA list of fortification permits for bakery products that are imported and sold in Norway [44]. We do not know how well the analytical samples, which are the basis of the present compilation, represent the total market of bread and bakery products, including the imported products. The average iodine content of bread and bakery products in our food composition database may therefore be too low, and therefore the estimations of iodine from bread may also be too low. Bread is a staple food in Norway, therefore new analyses of iodine in bread, both produced in Norway and imported products are warranted. 

Up until recently, analytical food composition iodine values have been scarce. Fortunately, new initiatives are being taken and we look forward to the development of more and higher quality iodine food composition data from both Norwegian and international initiatives. Comprehensive coverage of iodine values will strengthen the intake estimations. We will continue to compile and update our iodine values in the future when new analytical values emerge from future analytical projects.

## 5. Conclusions

Based on the last national dietary survey and newly updated and compiled iodine food composition data, we have shown that parts of the female Norwegian population and in particular young women have high probability of inadequate iodine intake. In men, almost a fifth had a high probability of inadequacy and a small group was in risk of too high intakes. In the context of differences in the consumption patterns of the iodine sources in different age groups future national dietary surveys, in children, adolescents and adults, and analysis of iodine in food and beverages in Norway are warranted. In addition, the iodine status of the population should be assessed using UIC. 

## Figures and Tables

**Table 1:** Average iodine content in selected food groups, µg/100 g.

	Iodine Content, µg/100 g		
Food Item	Mean	Min	Max
Bread	3	0	21
Cheese			
Cheese, whey	203	45	504
Cheese, white	27	6	79
Cheese, from goat milk	70	25	104
Coffee	8	3	15
Egg	36	34	38
Fish			
Atlantic cod ^#^	180	22	720
Saithe ^#^	219	35	820
Salmon, wild ^#^	20	7	75
Salmon, farmed ^#^	3.8	<1	9.1
Fruit and vegetables	0.7	0.1	5
Meat	4	0.2	43
Milk	16	15	20

^#^ Data from the Seafood Database, Institute of Marine Research and Nerhus et al. 2018 [16].

**Table 2:** Intakes of iodine in μg/day for men and women, in different age groups and according to reference intakes.

			Percentage of Subjects with Intakes			
Norkost 3 Population	Median	25th–75th Perc.	below LI (70 µg/day)	below AR (100 µg/day)	above RI (150 µg/day)	above UL (600 µg/day)
Men, all, *n* = 862	176	113–285	6.5	18.6	60.1	5.0
Men, 18–29 years, *n* = 138	153	99–233	7.3	25.4	53.6	2.9
Men, 30–39 years, *n* = 136	165	109–299	10.3	17.7	53.7	2.2
Men, 40–49 years, *n* = 179	164	110–248	5.0	21.2	59.8	3.4
Men, 50–59 years, *n* = 192	197	116–317	6.3	19.3	65.1	7.8
Men, 60–70 years, *n* = 217	191	127–352	5.1	12.0	64.1	6.9
Women, all, *n* = 925	130	88–215	12.8	32.9	41.4	1.1
Women, 18–29 years, *n* = 143	110	71–170	23.8	45.5	29.4	0
Women, 30–39 years, *n* = 169	125	86–184	12.4	35.5	37.9	0
Women, 40–49 years, *n* = 256	130	93–192	10.6	29.3	39.8	0.8
Women, 50–59 years, *n* = 193	159	95–265	11.9	30.1	52.3	2.1
Women, 60–70 years, *n* = 164	142	95–269	8.5	28.1	45.1	2.4

Intakes are given in median and 25th and 75th percentiles. LI, lower intake level; AR, average requirement; RI, recommended intake; UL, upper intake level [20].

**Table 3:** Percentage iodine intake from different food categories, in men, different age groups and for the consumers only, mean, and SD.

	Percentage Iodine Intake						
Food Categories	All, *n* = 862	Age 18–29, *n* = 138	Age 3049, *n* = 315	Age 50–70, *n* = 409	Consum.only	Nr.Consum.	*p* ^#^
Bread, cereals and cakes	6.0 (6.2)	7.3 (7.9)	6.1 (6.2)	5.4 (5.4)	6.0 (6.2)	860	<0.01
Fruit, berries, nuts and seeds	2.2 (2.6)	2.2 (2.5)	2.3 (2.8)	2.2 (2.5)	2.5 (2.6)	786	0.65
Vegetables and potatoes	2.3 (2.7)	2.6 (3.0)	2.3 (2.8)	2.2 (2.5)	2.3 (2.7)	848	0.27
Meat	3.4 (4.5)	4.9 (6.8)	3.4 (3.9)	2.9 (3.9)	3.5 (4.5)	848	<0.01
Fish, fish products and other seafood	21.8 (28.3)	10.5 (19.6)	19.2 (25.5)	27.7 (31.3)	32.7 (29.1)	576	<0.01
Lean fish	14.2 (26.9)	6.6 (18.5)	10.7 (23.6)	19.5 (30.3)	58.4 (19.5)	210	<0.01
Fat fish	0.5 (1.8)	0.2 (0.8)	0.5 (1.7)	0.7 (2.1)	3.2 (3.5)	140	<0.01
Egg	5.1 (8.5)	6.0 (10.1)	4.7 (8.1)	5.2 (8.2)	10.9 (9.5)	407	0.55
Milk, cheese and other dairy products ^§^	37.2 (22.6)	45.2 (22.5)	38.3 (22.5)	33.6 (21.9)	37.7 (22.2)	850	<0.01
Milk	21.5 (20.5)	26.9 (22.4)	22.5 (20.2)	19.0 (19.6)	27.3 (19.3)	680	<0.01
Yoghurt	2.3 (5.9)	2.1 (4.9)	2.6 (7.1)	2.2 (5.2)	9.8 (8.7)	202	1.00
Cheese, white	6.4 (8.4)	10.5 (11.7)	7.1 (7.4)	4.5 (6.3)	8.0 (8.7)	693	<0.01
Cheese, whey	5.4 (10.5)	4.5 (10.2)	4.7 (9.3)	6.1 (11.3)	16.8 (12.3)	275	<0.01
Beverages	12.9 (11.6)	9.3 (10.1)	13.9 (12.4)	13.4 (11.3)	12.9 (11.6)	862	<0.01
Mineral and drinking water	1.1 (1.3)	1.7 (1.8)	1.2 (1.3)	0.8 (0.9)	1.2 (1.3)	824	<0.01
Coffee	10.8 (11.3)	6.7 (9.9)	11.8 (11.9)	11.5 (10.9)	13.0 (11.1)	720	<0.01
Supplements	3.6 (11.9)	4.7 (12.2)	3.5 (12.7)	3.3 (11.2)	33.8 (17.7)	92	0.02

Data presented as mean and standard deviation (SD) although most of the data showed skewed distributions; ^§^ Dairy products not including butter; ^#^ Kruskal-Wallis test between the 3 age groups; “Consum.only” is the percentage intake of iodine from that food group for those who had an intake of that particular food group; “Nr.Consum” is the number of consumers of each food group.

**Table 4:** Percentage iodine intake from different food categories, in women, different age groups and for the consumers only, mean, and SD.

	Percentage Iodine Intake						
Food Categories	All, *n* = 925	Age 18–29, *n* = 143	Age 30–49, *n* = 425	Age 50–70, *n* = 357	Consum.only	Nr.Consum.	*p* ^#^
Bread, cereals and cakes	6.4 (6.6)	7.1 (6.3)	6.6 (6.9)	5.8 (6.3)	6.4 (6.6)	925	<0.01
Fruit, berries, nuts and seeds	3.2 (3.7)	3.5 (4.0)	3.2 (3.8)	3.1 (3.6)	3.4 (3.8)	886	0.89
Vegetables and potatoes	2.4 (2.7)	2.9 (3.3)	2.4 (2.6)	2.2 (2.6)	2.4 (2.7)	917	0.02
Meat	2.6 (3.9)	3.8 (5.7)	2.4 (3.1)	2.4 (3.8)	2.7 (3.9)	888	<0.01
Fish and other seafood	20.2 (27.4)	13.0 (22.0)	17.8 (25.9)	25.9 (30.0)	32.2 (28.5)	581	<0.01
Lean fish	13.7 (26.2)	8.5 (21.3)	11.5 (24.2)	18.3 (29.3)	57.5 (19.0)	220	<0.01
Fat fish	0.5 (1.5)	0.7 (2.2)	0.4 (1.4)	0.5 (1.4)	2.9 (2.8)	150	0.08
Egg	5.3 (8.1)	4.1 (7.4)	5.3 (8.3)	5.9 (8.2)	11.1 (8.5)	447	<0.01
Milk, cheese and other dairy products ^§^	33.8 (21.7)	42.4 (22.7)	34.1 (21.0)	30.0 (21.3)	34.1 (21.6)	916	<0.01
Milk	14.5 (17.6)	20.3 (20.5)	14.2 (16.6)	12.5 (17.0)	21.8 (17.5)	615	<0.01
Yoghurt	3.8(7.7)	4.6 (9.4)	3.7 (7.3)	3.5 (7.5)	11.4 (9.7)	306	0.80
Cheese, white	7.0 (7.8)	9.7 (10.4)	7.2 (7.6)	5.6 (6.5)	8.1 (7.9)	797	<0.01
Cheese, whey	6.3 (12.2)	5.6 (12.9)	6.8 (12.6)	6.1 (11.4)	18.4 (14.6)	317	0.12
Beverages	15.0 (12.4)	11.4 (11.1)	16.5 (13.0)	14.8 (11.9)	15.0 (12.4)	925	<0.01
Mineral and drinking water	1.7 (1.9)	2.5 (2.6)	1.7 (1.8)	1.4 (1.5)	1.7 (1.9)	925	<0.01
Coffee	11.5 (12.0)	7.5 (10.7)	12.9 (12.7)	11.6 (11.4)	14.6 (11.8)	731	<0.01
Supplements	6.1 (16.1)	4.8 (13.1)	6.6 (17.2)	6.0 (15.8)	38.7 (19.7)	145	0.78

Data presented as mean and standard deviation (SD) although most of the data showed skewed distributions; ^§^ Dairy products not including butter; ^#^ Kruskal-Wallis test between the 3 age groups; “Consum.only” is the percentage intake of iodine from that food group for those who had an intake of that particular food group; “Nr.Consum.” is the number of consumers of each food group.
